# Naa10p and IKKα interaction regulates EMT in oral squamous cell carcinoma via TGF‐β1/Smad pathway

**DOI:** 10.1111/jcmm.16680

**Published:** 2021-05-31

**Authors:** Sai Lv, Ting Luo, Yongyong Yang, Yuqing Li, Jie Yang, Jiang Xu, Jun Zheng, Yan Zeng

**Affiliations:** ^1^ Key Laboratory of Xinjiang Endemic and Ethnic Disease School of Medicine Shihezi University Shihezi China; ^2^ Department of Stomatology The First Affiliated Hospital School of Medicine Shihezi University Shihezi China; ^3^ Department of Urology Northwestern University Feinberg School of Medicine Chicago IL USA; ^4^ Department of Urological Surgery The Third Affiliated Hospital of Shenzhen University Shenzhen University Shenzhen China

**Keywords:** EMT, IKKα, Naa10p, OSCC, TGF‐β1/Smad

## Abstract

Epithelial‐mesenchymal transition (EMT) has been contributed to increase migration and invasion of cancer cells. However, the correlate of Naa10p and IKKα with EMT in oral squamous cell carcinoma (OSCC) is not yet fully understood. In our present study, we found N‐α‐acetyltransferase 10 protein (Naa10p) and IκB kinase α (IKKα) were abnormally abundant in oral squamous cell carcinoma (OSCC). Bioinformatic results indicate that the expression of Naa10p and IKKα is correlated with TGF‐β1/Smad and EMT‐related molecules. The Transwell migration, invasion, qRT‐PCR and Western blot assay indicated that Naa10p repressed OSCC cell migration, invasion and EMT, whereas IKKα promoted TGF‐β1–mediated OSCC cell migration, invasion and EMT. Mechanistically, Naa10p inhibited IKKα activation of Smad3 through the interaction with IKKα directly in OSCC cells after TGF‐β1 stimulation. Notably, knockdown of Naa10p reversed the IKKα‐induced change in the migration, invasion and EMT‐related molecules in OSCC cells after TGF‐β1 stimulation. These findings suggest that Naa10p interacted with IKKα mediates EMT in OSCC cells through TGF‐β1/Smad, a novel pathway for preventing OSCC.

## 1 INTRODUCTION

Oral cancer is one of the most common cancers in the world, and oral squamous cell carcinoma (OSCC) accounts for more than 90% of all oral malignancies.^1^, ^2^, ^3^, ^4^ Accumulating studies have shown that there is a close relation between the epithelial‐mesenchymal transition (EMT) and cancer progression.^5^, ^6^, ^7^ EMT is the conversion of cells from epithelial phenotype to mesenchymal phenotype.^8^ Cancer cells undergoing EMT display increased migratory and invasive properties.^9^ TGF‐β signalling pathways have been reported to be involved in EMT.^10^ Transforming growth factor (TGF)‐β1 is a crucial driver of EMT, regulates the transcription of downstream target genes and activates downstream signalling pathways to induce EMT.^11^ Although a large number of studies have made progress in illuminating the mechanism of EMT programming, the specific molecular mechanism of TGF‐β1–mediated EMT during OSCC deserves further investigation.

N‐α‐acetyltransferase 10 protein (Naa10p), also known as arrest defective 1 (ARD1), is the catalytic subunit of N‐acetyltransferase A that catalyses N‐α‐acetylation and ε‐acetylation.^12^, ^13^, ^14^, ^15^ Naa10p regulates a wide range of cellular functions, including autophagy,^16^ cell proliferation,^17^ apoptosis,^18^ cell cycle^13^ and cell motility.^19^ Our previous study indicated that Naa10p is highly expressed in OSCC tissues and the level of Naa10p inversely correlates with TNM stage, lymph node status, differentiation and recurrence, and Naa10p also inhibits migration and invasion in OSCC cells.^20^ However, the correlation between Naa10p expression and EMT has not been reported, not to mention in OSCC. Hence, we focused on the relationship between Naa10p and EMT.

IKK and NF‐κB signalling has been participated in promoting tumour cell survival, inflammatory and angiogenesis responses.^21^ IκB kinase α (IKKα) is a catalytic component of the IKK complex, which is a key switch in co‐ordinating both NF‐κB–dependent and NF‐κB–independent gene transcription.^22^, ^23^ IKKα can promote tumorigenesis via p27 in HER2‐positive epithelial cells.^24^ In addition, up‐regulated expression of IKKα can promote ovarian cancer epithelial cell proliferation, migration and invasion.^25^ Furthermore, TGF‐β1/Smad signalling is controlled by IKKα in the Panc1 cells and human breast cancer cell line MDA‐MB231.^26^, ^27^ Our previous research found that IKKα is highly expressed in OSCC tissues and the level of IKKα correlates with lymph node status and prognosis, and IKKα also promotes migration and invasion in OSCC cells.^28^ As both Naa10p and IKKα are involved in the migration and invasion of OSCC cells, are they related to the EMT and TGF‐β1/Smad pathways?

In this study, the related mechanisms of Naa10p and IKKα in OSCC were investigated. The Cancer Genome Atlas (TCGA) databases were used to predict the correlation of Naa10p and IKKα expression with TGF‐β1/Smad pathway and EMT‐related factors in OSCC. Next, in vitro experiments showed that Naa10p and IKKα were related to migration and invasion of OSCC cells, and the expression of EMT markers. Finally, we confirmed that Naa10p interacted with IKKα to regulate EMT of OSCC via TGF‐β1/Smad signalling pathways.

## 2 MATERIALS AND METHODS

### 2.1 Patients and samples

The collection of tissue samples from OSCC patients was approved and supervised by the Research Ethics Committee of the First Affiliated Hospital of the Medical College, Shihezi University. All tumours were classified in line with the staging criteria in the proposed eighth edition TNM classification system, including 72 stage I/II tumours and 22 stage III/IV tumours. Overall survival (OS) was calculated from the date of surgery to the date of death irrespective of the cause. Disease‐free survival (DFS) time was calculated from the date of surgery to the date of relapse. No patients received preoperative chemotherapy or radiotherapy, and all underwent radical surgery. The follow‐up period was up to 8 years by interviews in clinic or by the phone.

### 2.2 Bioinformatic analysis

The TCGA RNA‐Seq from TCGA website (https://cancergenome.nih.gov/) and the Gene Expression Omnibus database (http://www.ncbi. nlm.nih.gov/geo[gse30784]) were applied to analyse the tumour EMT scores^29^ and expression of Naa10p and IKKα, and the TCGA database also was used to explore the relation between Naa10p, IKKα and TGF‐β‐Smad, and EMT‐related molecules in OSCC.

### 2.3 Cell culture

Human OSCC cell line TCA8113 was purchased from China Center for Type Culture Collection, CAL27, SCC9, SCC15 and SCC25 cells were obtained from the American Type Culture Collection (ATCC). All these cells were cultured in Dulbecco's modified Eagle's medium (DMEM, Gibco) supplemented with 10% foetal bovine serum (FBS, HyClone) and penicillin‐streptomycin (Thermo Scientific). Normal oral epithelial cells (HOK) were purchased from BeNa Culture Collection (Beijing, China) and cultured in Minimum Essential Medium (MEM, Gibco) supplemented with 10% foetal bovine serum (FBS, HyClone) and penicillin‐streptomycin (Thermo Scientific).

### 2.4 Cell transfection

IKKα small interfering RNA (siRNA: 5′‐GTCTTGTCGCCTAGAGCTAdtdt‐3′) and negative control (5′‐CAGTCGCGTTTGCGACTGGdtdt‐3′) were synthesized by Sangon Biotech (Shanghai, China). Targeted short hairpin RNA knocking down Naa10p ( 5′‐CCCUGCACCUCUAUUCCAA‐3′) and control short hairpin RNA sequence (5′‐UUCUCCGAACGUGUCACGU‐3′) were inserted into psilencer2.1‐U6/neo plasmid. Cell transfection was conducted according to the instructions of Lipofectamine 2000 (Invitrogen) and cultured for 24‐48 hours for subsequent experimentation. Quantitative real‐time PCR (qRT‐PCR) and Western blot analysis were adopted to detect the knockdown efficiency.

### 2.5 Quantitative real‐time PCR

Total RNA from the frozen tissues and cells was extracted using TRIzol reagent (Invitrogen), and RNA was reversely transcribed into cDNA following the instructions of PrimeScript™ RT Reagent Kit and cDNA Synthesis Kit (Takara). In order to quantify mRNA levels of various genes, qRT‐PCR was performed with SYBR Green qPCR Master Mix (TOYOBO) and glyceraldehyde‐3‐phosphate dehydrogenase (GAPDH) mRNA was amplified as an internal control. The primers sequences were as follows: Naa10p (forward: ATGAACATCCGCAATG, reverse: ACAATCTTCCCATTCTC), E‐cadherin (forward: TACGCCTGGGACTCCACCTA, reverse: CCAGAAACGGAGGCCTGAT), Snail (forward: CCACACTGGCGAGAAG, reverse: AGAAGGTCCGAGCACAC), Slug (forward: TTTGCAAGATCTGCGGCAAG, reverse: CTGCAAATGCTCTGTTGCAGTG) and Claudin‐1 (forward: TCACTCCCAGGAGGATGC, reverse: GGCAGATCCAGTGCAAAGTC).

### 2.6 Western blot analysis

Total protein from OSCC tissue samples and cells was extracted using RIPA lysis buffer containing 1% protease inhibitor (Sigma). 50ug of protein was loaded on a 10% sodium dodecyl sulphate‐polyacrylamide gel electrophoresis and separated by electrophoresis. Then, the protein was transferred to polyvinylidene fluoride membranes (Merck Millipore). After blocking non‐specific binding sites with 5% non‐fat milk, the membranes were incubated with the following primary antibodies: anti‐IKKα (sc‐7606, 1:500; Santa Cruz Biotechnology), anti‐Naa10p (sc‐3739201:1000; Santa Cruz), anti–E‐cadherin (9782, 1:1000; Cell Signaling Technology, CST), anti‐Snail (9782, 1:1000; CST), anti‐Slug (9782, 1:1000; CST), anti–Claudin‐1 (9782, 1:1000; CST), anti‐Smad2 (5339, 1:1000; CST), anti–p‐Smad2 (3108, 1:1000; CST), anti‐Smad3 (9523, 1:1000; CST), anti–p‐Smad3 (9520, 1:1000; CST), anti–β‐Actin (TA09, 1:1000; ZSGB‐BIO, Beijing, China) overnight at 4℃. Next, the membranes were washed with TBST and incubated with peroxidase‐conjugated IgG antibody at room temperature for 2 hours, and the immune complex was visualized by using enhanced chemiluminescence (34094; Pierce).

### 2.7 Cell migration and invasion assay

The cell migration and invasion assays were performed in the 24‐well Transwell plates (0.8‐m pore size filter; 3422; Corning) as follows: CALl27 and SCC15 cells were transfected with shNaa10p or IKKα siRNA for 24 hours with or without TGF‐β1 treatment, and 5 × 10^4^ cells suspended in 200 µL serum‐free medium were added to the upper chamber. The lower chamber was filled with 800 µL DMEM culture medium containing 10% FBS (04‐001; Biological Industries, Kibbutz Beit HaEmek). After 24 hours of incubation at 37°C, cells that moved to the bottom surface of the chamber were fixed with 100% methanol for 30 minutes and then stained with 0.5% crystal violet and the cell number was counted under a microscope in nine different fields/filter. The invasion studies were performed as the migration assay except that the upper chambers of 24‐well Transwell plates were coated with 100μL Matrigel (1 mg/mL) (356234; BD Biosciences) at 37°C for 3‐4 hours.

### 2.8 Immunofluorescence

CAL27 cells seeded on coverslips were exposed to TGF‐β1(10 ng/ml) (ProteinTech) in culture medium for 12 hours. After washing with PBS for three times, cells were fixed in 4% paraformaldehyde for 30 minutes, permeabilized by 0.5% Triton ×‐100 for 10 minutes and blocked in 5% bovine serum albumin for 30 minutes. The cells then were incubated with specific primary antibodies against Naa10p and IKKα overnight at 4°C. Then, slides were incubated with Fluorescein‐Conjugated Goat anti‐Mouse IgG (ZF‐0312; ProteinTech Group Inc) and Rhodamine‐Conjugated Goat anti‐Rabbit IgG (ZF‐0316; ProteinTech Group Inc) for 1 hour at room temperature and the nuclei were stained with 4′,6‐diamidino‐2‐phenylindole. Images were captured using an Olympus IX‐71 fluorescence inverted microscope (Leica Microsystems).

### 2.9 Immunoprecipitation assay

The CAL27 or SCC15 cells were incubated in weak‐potency RIPA lysis buffer for 30 minutes at 4°C and then centrifuged at 12 000 × *g* for 20 minutes. Supernatants were incubated with protein A/G beads (Santa Cruz Biotechnology) overnight at 4°C to pre‐clear the lysates. The pre‐cleared lysates were incubated at 4°C with anti‐Naa10p or control IgG (ZSGB‐BIO) and protein A/G overnight according to the manufacturer's instructions. The protein A/G‐antibody‐antigen complex was concentrated by centrifugation at 1000 × *g* for 10 minutes at 4°C and washed with PBS three times. After washing, the complexes were boiled in 2 × SDS loading buffer and loaded for Western blot detection.

### 2.10 Glutathione S‐transferase protein pull‐down assay

GST‐pGEX‐4T1‐Naa10p was transformed into Escherichia coli and induced for the expression of GST or GST fusion protein by IPTG. Proteins were purified using the Beaver Beads TM GSH Kit (Beaver Biosciences Inc) according to the manufacturer's instructions. Pull‐down assays were performed by incubating GST fusion protein with the cell lysates of CAL27 cells, which were transfected with His‐IKKα at 4°C for 12 hours. Then, the bead‐bound protein complexes were then washed, boiled in 2 × SDS loading buffer and loaded for Western blot detection.

### 2.11 Luciferase assay

For the luciferase reporter assay, CAL27 and HEK293T cells in 24‐well plates were co‐transfected with 0.5μg of pGL3‐SMAD3, 0.05 μg of pRL‐SV40 (Promega) and 0.5 μg of control DNA using Lipofectamine 2000. After 24 hours, cells were treated with TGF‐β1 (5 ng/ml) for 6 hours. Luciferase activities were measured using the Dual‐Luciferase Reporter Assay System (Promega) according to the manufacturer's instructions.

### 2.12 Statistical analysis

All the analyses were carried out by SPSS 20.0 (IBM SPSS). Data were presented as the mean ±standard deviation. A Pearson correlation index was used to estimate the correlation between Naa10p, IKKα and TGF‐β‐Smad, and EMT‐related molecules. The Kaplan‐Meier survival curves were used to evaluate the survival of patients. A two‐tailed Student's *t* test was used to determine significant differences between two groups. *P* < .05 was considered statistically significant.

## 3 RESULTS

### 3.1 EMT participated in the development of OSCC

**FIGURE 1:**
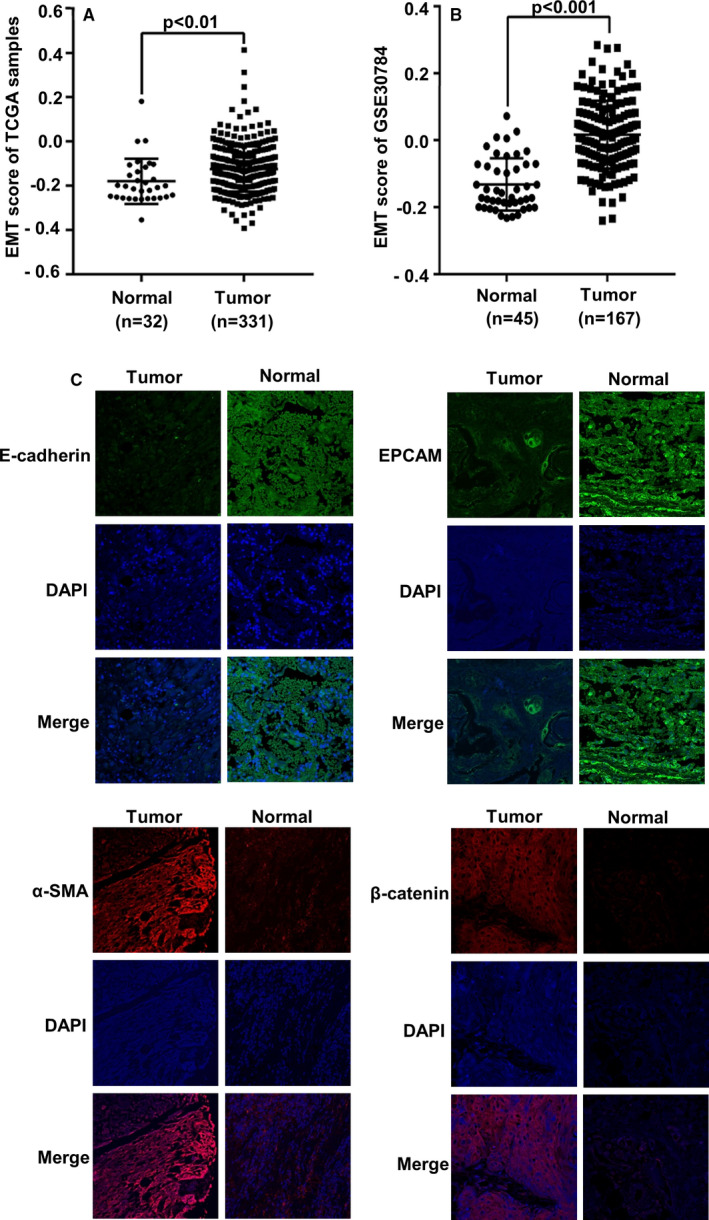
EMT is closely related to OSCC. A, EMT scores of normal (n = 32) and OSCC (n = 331) tissues in the TCGA database. B, EMT scores of normal (n = 45) and OSCC (n = 167) tissues in the GEO database. C, Immunofluorescence (IF) staining was used to examine the expression of epithelial markers E‐cadherin and EPCAM, and mesenchymal markers α‐SMA and β‐catenin

To determine the relationship between EMT and OSCC, we explored the EMT scores in normal and OSCC tissues with RNAseq data from the TCGA and GEO databases. Compared with the normal tissues, OSCC tissues had a high EMT score (Figure 1A, B). Meanwhile, immunofluorescence results showed that the expression of epithelial markers, E‐cadherin and EPCAM, was higher in normal tissues than in OSCC tissues, whereas the expression of mesenchymal markers, α‐SMA and β‐catenin, was lower in normal tissues than in OSCC tissues (Figure 1C).

### 3.2 Naa10p and IKKα were overexpressed in OSCC and related to EMT and prognosis

**FIGURE 2:**
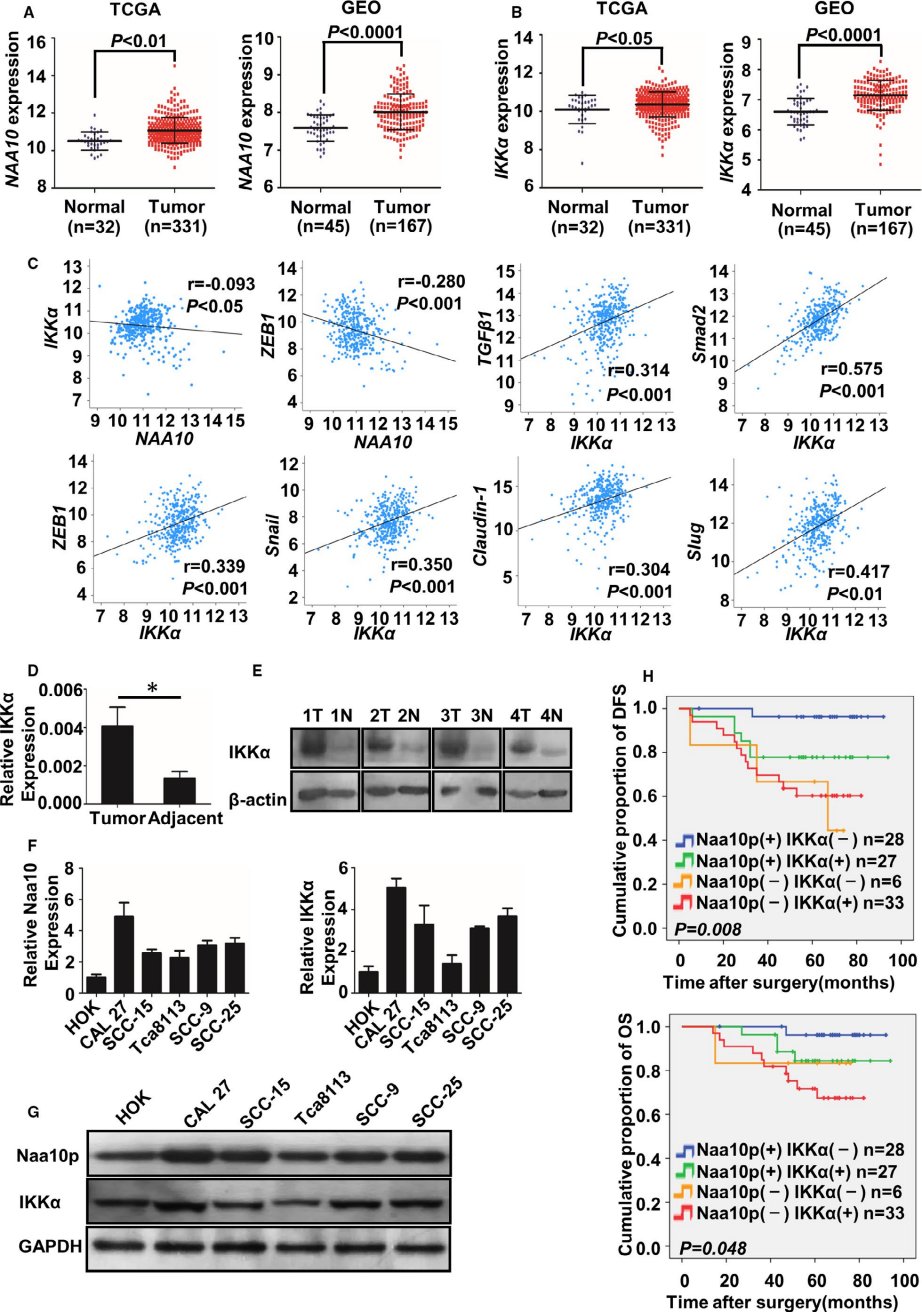
Expressions of Naa10p and IKKα were related to TGF‐β/Smad, EMT‐related gene and survival prognosis. A, B, TCGA and GEO showed the expression of Naa10p and IKKα in adjacent normal and OSCC tissues. C, The correlation of Naa10p with IKKα, Naa10p with ZEB1 and IKKα with TGF‐β1, Smad2, ZEB1, Snail, Claudin‐1 and Slug analysed by the Pearson correlation analysis. D, IKKα mRNA expression in OSCC tissues and paired adjacent normal tissues by quantitative real‐time polymerase chain reaction (qRT‐PCR). The logarithmic scale of 2‐^ΔΔCt^ was used to measure the fold change. GAPDH as internal reference. E, Western blot was performed to check IKKα protein expression in four paired OSCC tissue samples, with β‐actin as loading control. F, G, Expression levels of Naa10p and IKKα were determined by qRT‐PCR and Western blotting in normal oral epithelial cells (HOK) and OSCC cell lines. H, Kaplan‐Meier analysis showed disease‐free survival (DFS) and overall survival (OS) of OSCC patients with Naa10p and IKKα expression. The data comparison of OSCC tissues and adjacent normal tissues was analysed by paired *t* test. *P < .05, N, adjacent normal tissue; T, tumour tissue

The expression of Naa10p and IKKα in OSCC tissues was analysed in the TCGA and GEO databases. The expression of Naa10p and IKKα was significantly higher in OSCC tissues than in adjacent tissues (Figure 2A, B). Besides, the expression of Naa10p was negatively correlated with the expression of IKKα and ZEB1 (*r* < 0, *P* < .05), whereas the expression of IKKα was positively correlated with the expression of IKKα and was positively correlated with the expression of TGF‐β1, Smad2, ZEB1, Snail, Claudin‐1 and Slug (*r* > 0, *P* < .05) (Figure 2C). Next, we detected IKKα mRNA and protein expression in 4 pairs of OSCC tissues and the adjacent normal tissues. IKKα expression was markedly higher in OSCC tissues compared with the adjacent normal tissues (Figure 2D, E, P <.05). Our previous study showed that Naa10p was overexpressed in OSCC tissues.^20^ Additionally, the expression levels of Naa10p and IKKα in five oral cancer cell lines (Tca8113, Cal‐27, SCC9, SCC15 and SCC25) and normal oral epithelial cells (HOK) were detected by qRT‐PCR and Western blot. As shown in Figure 2F, G, the expression levels of Naa10p and IKKα were much higher in oral cancer cells than in normal oral epithelial cells. These data suggested that Naa10p and IKKα expression was higher in cancerous tissues and oral cancer cells. Moreover, patients with high expression of Naa10p had a good prognosis,^30^ whereas those with low expression of IKKα had a good prognosis.^28^ Our study found that patients with high Naa10p and low IKKα had the best prognosis (Figure 2H).

### 3.3 Knockdown of Naa10p promoted OSCC migration, invasion and EMT

**FIGURE 3:**
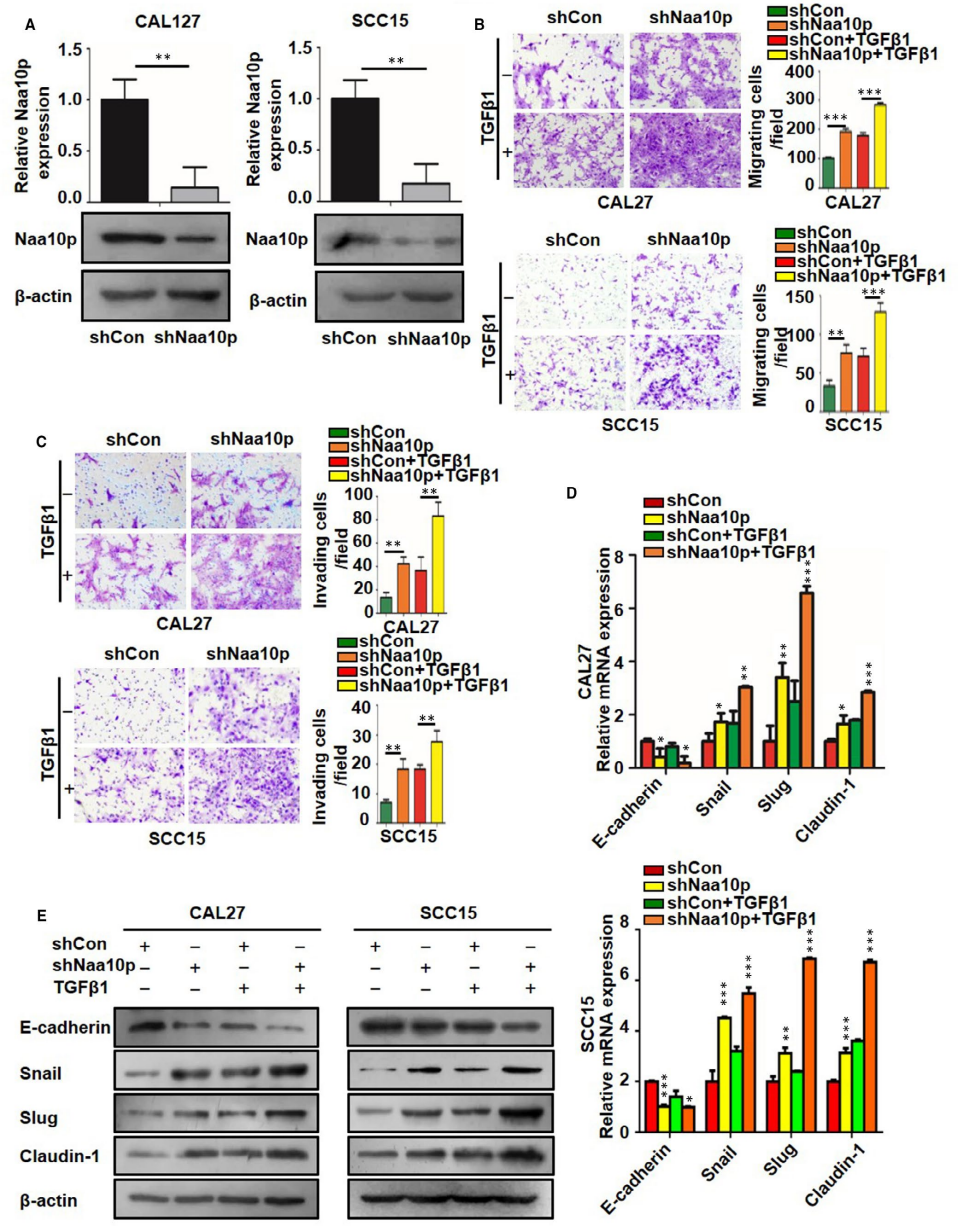
A, qRT‐PCR and Western blot analysis of Naa10p in CAL27 and SCC15 cells transfected with indicated constructs. B, C, Migration and invasion analysis of CAL27 and SCC15 cells transfected with control (shCon) or Naa10p shRNA (shNaa10p) with or without TGF‐β1 treatment. D, qRT‐PCR analysis of E‐cadherin, Snail, Slug and Claudin‐1 mRNA levels in CAL27 and SCC15 cells transfected with indicated constructs after 48 h and then stimulated with TGF‐β1. E, Western blot analysis of E‐cadherin, Snail, Slug and Claudin‐1 protein levels in CAL27 and SCC15 cells treated as in D. Mean fold change was calculated from three independent experiments performed in triplicate, *P < .05, ** *P* < .01, *** *P* < .001

To verify whether Naa10p is necessary for suppressed OSCC, endogenous Naa10p expression was silenced in CAL27 and SCC15 cells. qRT‐PCR and Western blot analysis proved a significant decrease in Naa10p expression in the shNaa10p group compared with the control group (Figure 3A, *P* < .05). Knockdown of Naa10p in OSCC cells significantly increased both cell migration and invasion capacity, as illustrated by migration and invasion assay (Figure 3B, C, *P* < .05). In addition, in order to confirm whether altering Naa10p mediated the EMT‐related molecular expression. We measured the levels of classical EMT markers by qRT‐PCR and Western blot in CAL27 and SCC15 cells after knocking down Naa10p. These cells had an increased abundance of mesenchymal biomarkers Snail, Slug and Claudin‐1, but reduced protein abundance of epithelial biomarker E‐cadherin. At the same time, we also found no difference after adding TGF‐β1 stimulation (Figure 3D, E, *P* < .05). These results indicated that the silencing Naa10p promoted the migration, invasion and EMT of OSCC cells in vitro.

### 3.4 Knockdown of IKKα inhibited OSCC migration, invasion and EMT in TGF‐β1–treated OSCC cells

**FIGURE 4:**
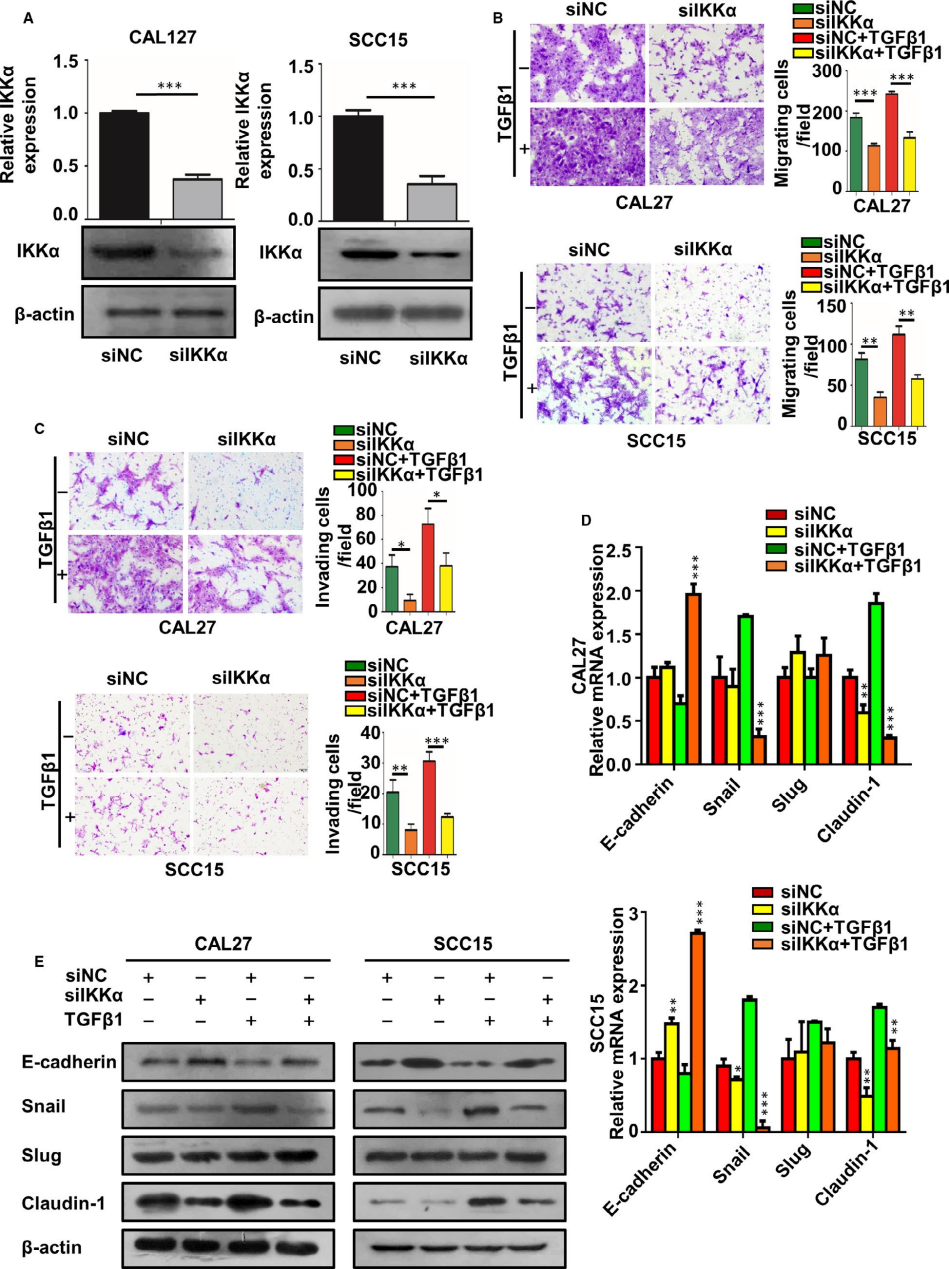
A, qRT‐PCR and Western blot analysis of IKKα in CAL27 and SCC15 cells transfected with control and IKKα siRNA. B, C, Transwell assay was conducted to explore the effect of IKKα and TGF‐β1 on migration and invasion of OSCC cells. The migrated and invaded cells were detected by crystal violet staining, and images were captured. D, E, CAL27 and SCC15 cells were transfected with control and IKKα siRNA with or without TGF‐β1 treatment, and the E‐cadherin, Snail, Slug and Claudin‐1 mRNA and protein levels were detected using qRT‐PCR and Western blot analysis. *P < .05, ** *P* < .01, *** *P* < .001

To investigate the influence of IKKα on OSCC cell migration and invasion, short interfering RNA (siRNA) targeting IKKα was transfected into CAL27 and SCC15 cells to knock down IKKα (Figure 4A, *P* < .05). Knocking down IKKα reduced cell migration and invasion. Moreover, knocking down IKKα suppressed TGF‐β1, which increased the migratory and invasive capacity in CAL27 and SCC15 cells (Figure 4B, C, *P* < .05). To further characterize the role of the IKKα in EMT, we measured the changes in EMT‐related markers after knocking down IKKα. As shown in Figure 4D, E, after being transfected with siIKKα and treatment with TGF‐β1, the expression of E‐cadherin is up‐regulated, whereas the expression of Snail and Claudin‐1 was down‐regulated (Figure 4D, E, *P* < .05).

### 3.5 Naa10p interacted with IKKα

**FIGURE 5:**
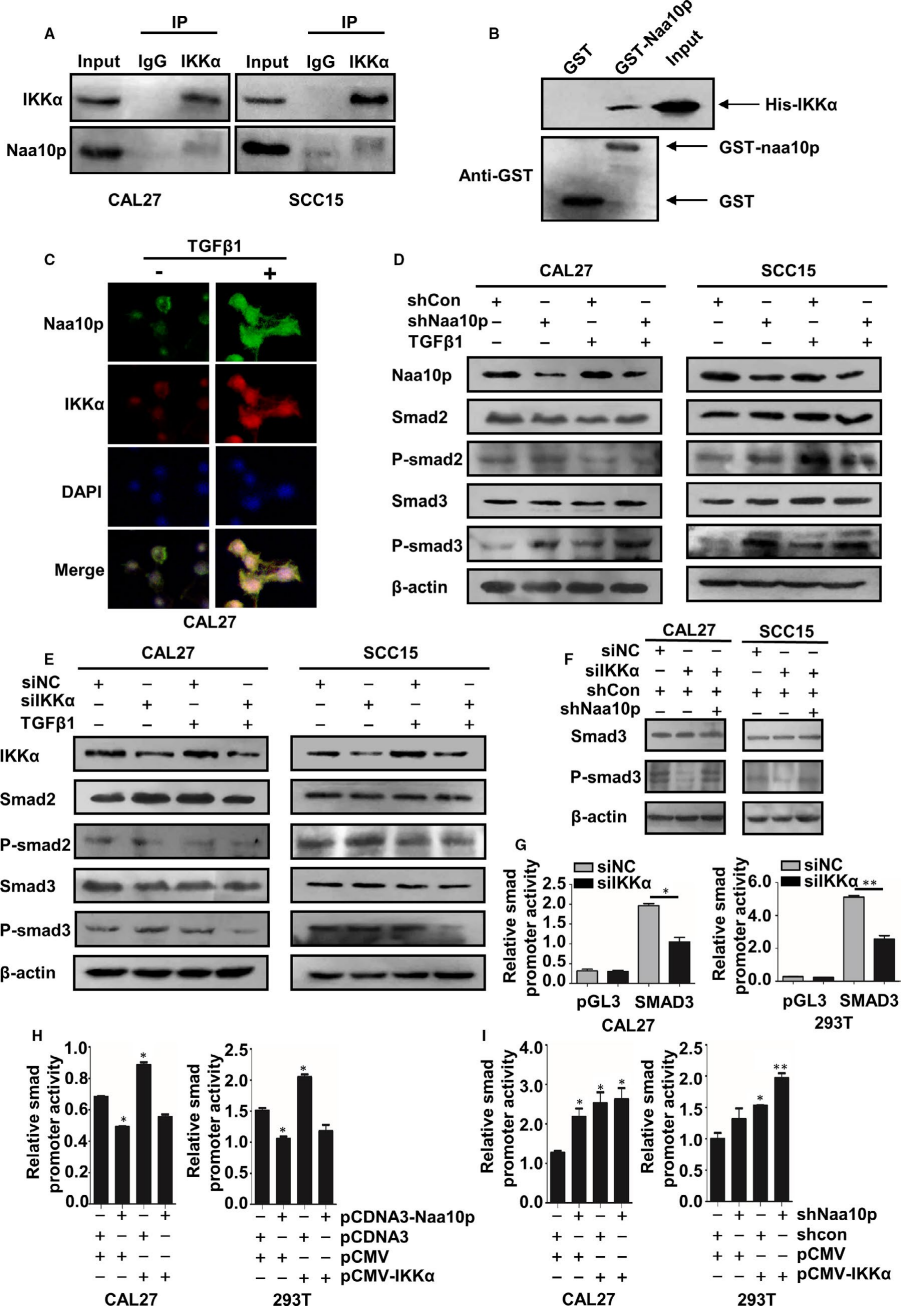
Naa10p interacts with IKKα and suppressed IKKα‐activated Smad3 signal pathway. A, Immunoprecipitation of Naa10p and IKKα with an anti‐IKKα antibody in CAL27 and SCC15 cells. B, GST pull‐down assay results indicated the direct interaction between Naa10p and IKKα. C, Laser scanning confocal microscopy (LSCM) technique was used to show the colocalization of Naa10p and IKKα in CAL27 cells. D, E, Western blot analysis for phosphorylation and expression of Smad2 and Smad3 in CAL27 and SCC15 cells infected with shNaa10p or siIKKα with or without TGF‐β1. F, Knockdown of Naa10p rescued the function of IKKα altering phosphorylated Smad3 (with TGF‐β1). G, H, I, CAL27 and SCC15 cells were transfected with the corresponding constructs for 24 h and further treated with TGF‐β1 for 6 h. The cell lysates were used to measure luciferase activity. *P < .05, ** *P* < .01, *** *P* < .001

In order to gain a better understanding of whether Naa10p interacted with IKKα in CAL27 and SCC15 cells, we performed immunoprecipitation experiments, which showed that Naa10p was pulled down by the anti‐IKKα antibody, indicating that endogenous Naa10p was physically associated with IKKα in CAL27 and SCC15 cells (Figure 5A). Moreover, a direct association between GST‐Naa10p and His‐IKKα was confirmed by GST pull‐down assays (Figure 5B). Immunofluorescence staining revealed that Naa10p and IKKα were co‐localized in both cell cytoplasm and nucleus. Additionally, TGF‐β1 treatment promoted the nuclear localization of Naa10p and IKKα (Figure 5C).

### 3.6 Naa10p suppressed IKKα‐activated Smad3 signalling pathway

IKKα participates in TGF‐β–induced EMT through a Smad‐dependent pathway.^26^ In order to verify whether IKKα plays a role through the TGF‐β1/Smad pathway in OSCC, TGF‐β1 stimulation was added at the time of transfection of siIKKα, and the level of phosphorylated Smad3 was obviously decreased, whereas the level of phosphorylated Smad2 was not affected (Figure 5E). We then found that knocking down Naa10p increased the phosphorylation levels of Smad3 in CAL27 and SCC15 cells (Figure 5D). However, after interference with Naa10p and IKKα at the same time, and under the TGF‐β1 stimulation, reduction in P‐Smad3 caused by interference with IKKα alone could be increased (Figure 5F). We investigated TGF‐β1–induced Smad3 activation using a Smad3‐dependent luciferase reporter gene construct. Here, a distinctly decreased activation of the Smad3 reporter was observed in cells transfected with IKKα‐specific siRNA and treated with TGF‐β1 (Figure 5G). Besides, the overexpression of IKKα enhanced Smad3’s promoter activity, but such activity was heightened by silencing Naa10p and reversed by co‐expressing Naa10p, indicating Naa10p could repress IKKα‐regulated Smad3 transcription (Figure 5H, I).

### 3.7 Naa10p can reverse the change in migration, invasion phenotype and EMT‐related factors caused by IKKα

**FIGURE 6:**
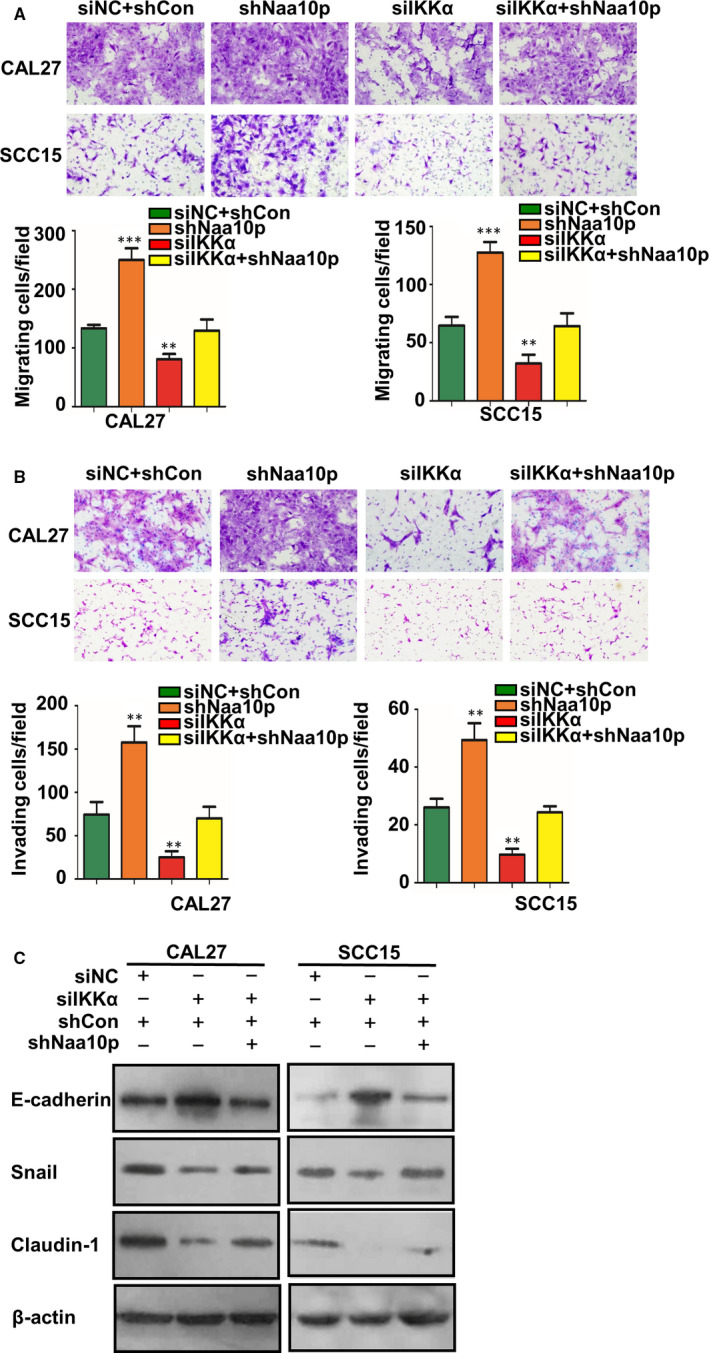
Downgrade of Naa10p blocks siIKKα‐induced OSCC cell phenotype and EMT. A, B, C Knockdown of Naa10p rescued the promotion effect of decreased IKKα on OSCC cell migration A, invasion B, ability and EMT C, and the beta‐actin bands in Figure 6C are identical to Figure 5F because of the same treatment on the same SDS‐PAGE gel. * *P* < .05, ** *P* < .01, *** *P* < .001

To confirm the role of the Naa10p‐IKKα‐Smad axis in TGF‐β1–mediated cell migration, invasion and EMT, we hypothesized that Naa10p knockdown would reverse the effects of IKKα knockdown in OSCC cells. We measured the migratory and invasive capacity of CAL27 and SCC15 cells, and cell migration and invasion were increased (Figure 6A, B, *P* < .05). In alignment with functional reversal, Western blot indicated the reversed expression of EMT markers occurred in CAL27 and SCC15 cells (Figure 6C, *P* < .05).

## 4 DISCUSSION

EMT is critical in regulating invasion and metastasis of malignant tumours. The expression of the epithelial markers during the EMT process such as E‐cadherin is down‐regulated, whereas that of the mesenchymal markers is increased.^31^ Our bioinformatic analysis results through the TCGA and GEO databases suggest a correlation between EMT and OSCC. There was a direct relation between EMT and OSCC, and our results indicated that the lower the expression of E‐cadherin and EPCAM, the higher the expression of α‐SMA and β‐catenin in OSCC tissue. Besides, we also found that there was a close correlation between Naa10p, IKKα expression and TGF‐β1/Smad pathway, and EMT‐related molecules by analysing RNAseq data in the TCGA database.

This study emphasized the essential role of Naa10p in tumour migration, invasion and EMT. Previous reports studied the role and function of Naa10p in various cancers, and it is widely believed that Naa10p inhibited the cell migration and invasion. Naa10p was found to suppress cell motility by inhibiting MLCK.^14^ Furthermore, Naa10p prevented the formation of GIT‐PIX‐paxillin complex by binding to GIT functional region of the PIX protein, reduced endogenous CDC42/Rac1 activity and thus inhibited cell migration.^19^ In addition, down‐regulation of Naa10p can promote the migration and invasion of breast cancer cells.^32^ These results are consistent with our findings in this study. Moreover, the expression of mesenchymal markers was up‐regulated, whereas epithelial markers were down‐regulated when Naa10p was knockdown, indicating that Naa10p inhibited EMT in OSCC cells.

Recent studies have revealed that IKKα could promote the invasion and metastasis capability of prostate cancer,^33^ breast cancer,^34^ ovarian cancer^25^ and pancreatic cancer.^26^ However, IKKα’s role in OSCC is still largely unknown. Our results showed that interfering IKKα significantly impaired the migration and invasion of OSCC cells. Although being transfected with siIKKα and stimulated with TGF‐β1, the cell migration and invasion ability were significantly reduced, and EMT‐related markers were also changed. These results indicate that IKKα was involved in the TGF‐β1–mediated cell migration, invasion and EMT. To some extent, this study further found that IKKα plays an important role in tumour migration and invasion.

TGF‐β signalling could induce EMT. Upon activation of the TGF‐β signalling pathway, TGF‐β1 interacts with the TβRII, which recruits and phosphorylates TβRI. Activated TβRI phosphorylates Smad2 and Smad3 at the C‐terminal serine residues. Subsequently, phosphorylated Smad2 and Smad3 form a complex with Smad4 and then translocate into the nucleus where they regulate the transcription of downstream EMT‐related genes.^35^, ^36^ However, how Naa10p and IKKα regulate EMT in OSCC has not been determined. IKKα was found to be related to TGF‐β‐Smad signalling pathway.^37^ Our results indicated Naa10p could interact with IKKα directly. Thus, we speculate that Naa10p/IKKα may regulate EMT through the TGF‐β/Smad pathway. As results shows, knocking down Naa10p enhanced the expression of phosphorylation of Smad3, whereas it did not affect the expression of total Smad2 and Smad3 and phosphorylation of Smad2. IKKα siRNA treatment inhibited TGF‐β1–induced phosphorylation of Smad3, whereas it did not affect the expression of total Smad2 and Smad3 and phosphorylation of Smad2. We performed luciferase assays, and the result revealed Naa10p could repress IKKα‐regulated Smad3 transcription. In addition, we uncovered that decreased Naa10p largely contributed to rescue the tumour‐suppressive phenotypes induced by down‐regulation of IKKα. These results suggest that Naa10p/IKKα modulates EMT through the TGF‐β1/Smad3 signalling pathway.

**FIGURE 7:**
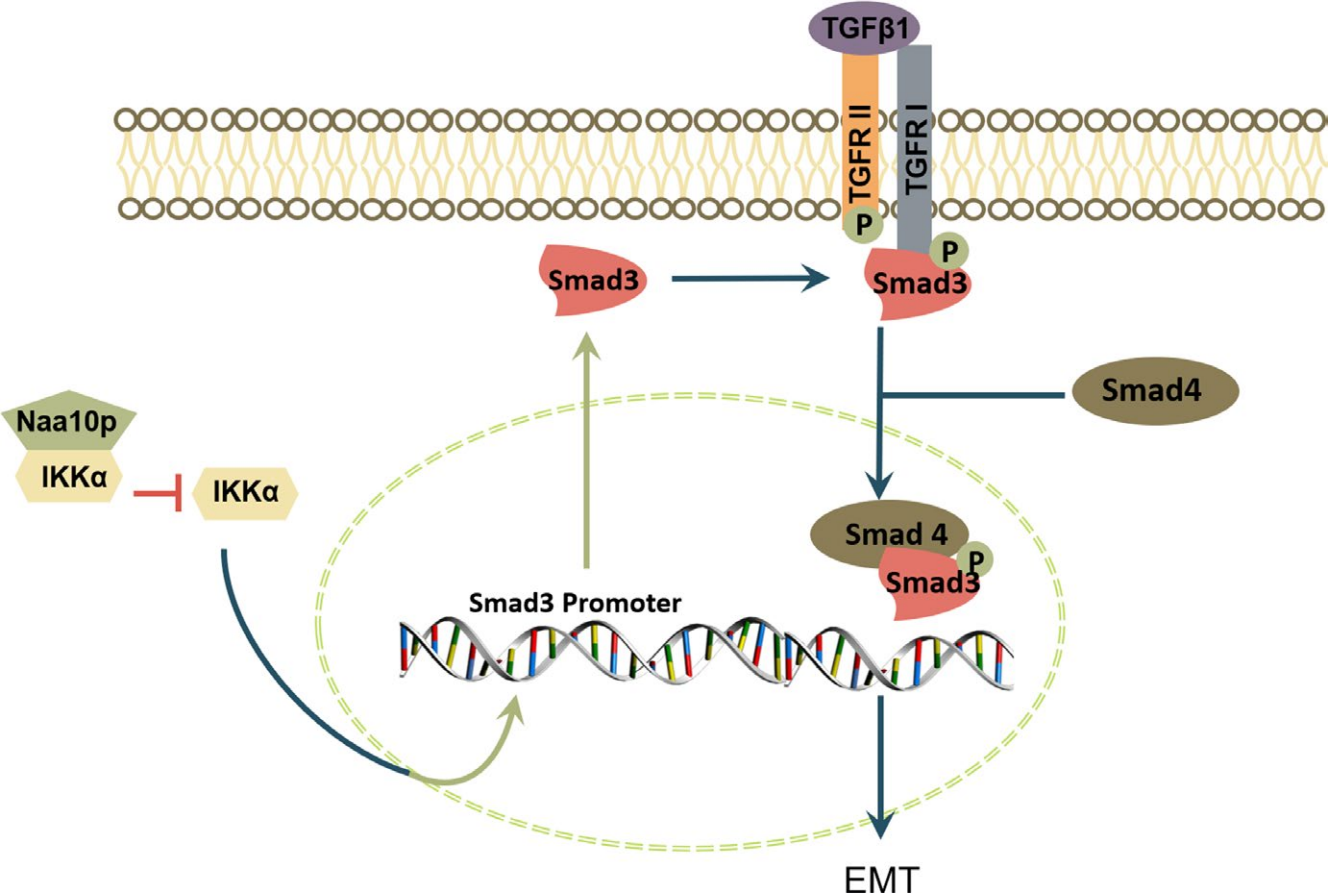
Schematic model indicates Naa10p interacts with IKKα to regulate EMT via TGF‐β1‐Smad3 signalling. TGF‐β1 induces EMT, Naa10p down‐regulates the transcriptional activation of Smad3 gene by IKKα through interaction with IKKα and leads to changes in the expression of downstream EMT‐related markers to regulate the migration and invasion of OSCC cells

In conclusion, this study investigated the potential role of Naa10p and IKKα in OSCC. Our data suggested that Naa10p can interact with IKKα to inhibit the activation of Smad3, sequentially repressed TGF‐β1/Smad signalling pathway to regulate EMT in OSCC (Figure 7). However, they regulate which EMT transcription factor was not validated, and the functions of Naa10p and IKKα in vivo needed future studies.

## CONFLICT OF INTEREST

The authors declare that they have no conflict of interests.

## AUTHOR CONTRIBUTIONS

**Sai Lv:** Data curation (lead); Formal analysis (lead); Writing‐original draft (lead). **Ting Luo:** Data curation (supporting); Formal analysis (equal); Writing‐original draft (lead). **Yongyong Yang:** Writing‐original draft (equal). **Yu qing Li:** Formal analysis (equal). **Jie Yang:** Resources (equal). **Jiang Xu:** Resources (lead). **Jun Zheng:** Project administration (equal); Resources (equal); Writing‐original draft (supporting). **Yan Zeng:** Project administration (lead); Writing‐original draft (equal).

## Data Availability

All data generated or analysed during this study are included in this published paper.
